# The Influence of Diet on MicroRNAs that Impact Cardiovascular Disease

**DOI:** 10.3390/molecules24081509

**Published:** 2019-04-17

**Authors:** Branislav Kura, Mihir Parikh, Jan Slezak, Grant N. Pierce

**Affiliations:** 1 Institute for Heart Research, Centre of Experimental Medicine, Slovak Academy of Sciences, 84104 Bratislava, Slovak Republic; branislav.kura@savba.sk (B.K.); jan.slezak@savba.sk (J.S.); 2 Institute of Cardiovascular Sciences and the Canadian Centre for Agri-food Research in Health and Medicine (CCARM), Albrechtsen Research Centre, St. Boniface Hospital, Winnipeg, MB R2H2A6, Canada; mparikh@sbrc.ca; 3 Department of Physiology and Pathophysiology, Faculty of Health Sciences, University of Manitoba, Winnipeg, MB R3E0W3, Canada

**Keywords:** bioactive food components, cardiovascular disease, heart, miRNA, nutrition

## Abstract

Food quality and nutritional habits strongly influence human health status. Extensive research has been conducted to confirm that foods rich in biologically active nutrients have a positive impact on the onset and development of different pathological processes, including cardiovascular diseases. However, the underlying mechanisms by which dietary compounds regulate cardiovascular function have not yet been fully clarified. A growing number of studies confirm that bioactive food components modulate various signaling pathways which are involved in heart physiology and pathology. Recent evidence indicates that microRNAs (miRNAs), small single-stranded RNA chains with a powerful ability to influence protein expression in the whole organism, have a significant role in the regulation of cardiovascular-related pathways. This review summarizes recent studies dealing with the impact of some biologically active nutrients like polyunsaturated fatty acids (PUFAs), vitamins E and D, dietary fiber, or selenium on the expression of many miRNAs, which are connected with cardiovascular diseases. Current research indicates that the expression levels of many cardiovascular-related miRNAs like miRNA-21, -30 family, -34, -155, or -199 can be altered by foods and dietary supplements in various animal and human disease models. Understanding the dietary modulation of miRNAs represents, therefore, an important field for further research. The acquired knowledge may be used in personalized nutritional prevention of cardiovascular disease or the treatment of cardiovascular disorders.

## 1. Introduction

Cardiovascular diseases (CVD) represent one of the most frequent causes of death worldwide. This has occurred despite the development of many different pharmaceutical substances to improve the lifespan, as well as the quality of life, of humans. The most likely explanation for this CVD morbidity despite the pharmacopeia of drugs available may be the unhealthy lifestyle patterns exhibited in most countries. Adherence to a regular diet of specific healthy nutrients, therefore, could be an effective strategy for prevention of CVD [1,2]. For example, people consuming the Mediterranean diet have a lower incidence of CVD [3,4]. Nutrients abundant in the Mediterranean diet like polyphenols, vitamins, dietary fiber, coenzyme Q10, polyunsaturated fatty acids (PUFAs) and minerals are thought to provide beneficial effects for many diseases, including CVD [3,4]. The molecular mechanism through which these bioactive nutrients produce their beneficial effects on CVD remains unclear.

MicroRNAs (miRNAs) are short RNA sequences belonging to the non-coding region of RNA [5]. MiRNAs have a significant effect on the expression of a wide range of proteins that will ultimately affect different molecular pathways [5]. Significant differences have been observed in the expression of many miRNAs in various diseases compared to healthy subjects [6,7]. As a result, miRNAs are considered to be potential biomarkers for many diseases as well as progressive therapeutic tools. Due to the impact of diet on CVD, research has begun to focus on the influence of diet on miRNA expression and the potential application of this information to therapeutic procedures in CVD [8].

The main goal of this review is to summarize recent information and studies concerned with the beneficial effects of bioactive dietary compounds on the cardiovascular system, with particular attention on the expression of different miRNAs. Understanding the mechanisms of action of nutrients through modulation of miRNA expression could be helpful in the prevention or treatment of diseases connected with the cardiovascular system.

## 2. Origin and Function of miRNAs

MicroRNAs belong to a group of non-coding RNAs which can exert a strong effect on gene expression post-transcriptionally by binding to the 3′ untranslated region (3′-UTR) of the target messenger RNA (mRNA) [9]. They are small (approximately 19–25 nucleotides long) RNA molecules and their binding to mRNA results in the inhibition of translation or mRNA degradation [10,11]. It is assumed that miRNAs are able to regulate at least 30% of the human protein-coding genome [12]. Interestingly, each miRNA can regulate several targets and more than one miRNA can function on a single mRNA. This suggests that miRNAs play a huge regulatory role in many biological processes like apoptosis, cell differentiation, cell proliferation or cell cycle progression [10,12,13,14]. The first miRNA, lin-4, was discovered by Ambros and colleagues in 1993 [15] and was isolated from *Caernohabditis elegans*. Presently, almost 2000 miRNAs have been identified in humans (http://www.miRbase.org – 7.3.2019). Approximately 150–200 of these have been found in the heart and were also connected with cardiovascular diseases [7].

**Figure 1:**
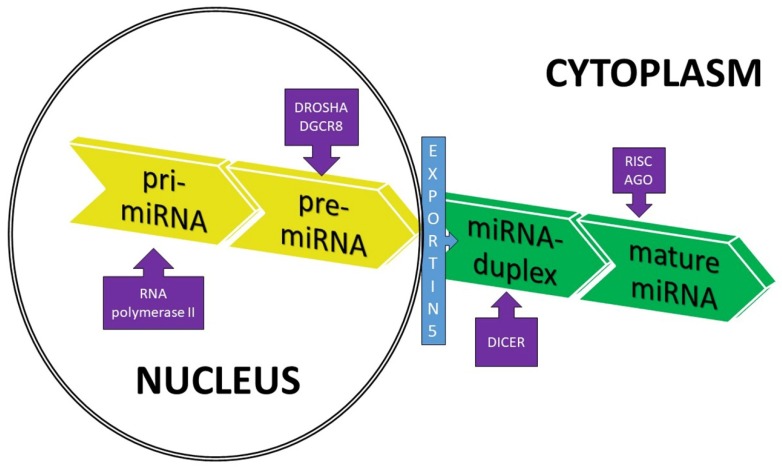
Schematic overview of microRNA (miRNA) biogenesis. DROSHA—RNase III enzyme; DGCR8—DiGeorge syndrome critical region 8; DICER—RNase III enzyme; RISC—RNA-induced silencing complex; and AGO—Argonaute protein.

The biogenesis of miRNA starts with the transcription of miRNA genes by RNA polymerase II (Figure 1). This process leads to the formation of a primary miRNA transcript—pri-miRNA— containing a cap structure at the 5′ end and a poly-adenylation at the 3′ end [16]. Pri-miRNA is then cleaved by a microprocessor complex, which consists of the double-stranded RNase III enzyme DROSHA and its essential cofactor—the DiGeorge syndrome critical region 8 (DGCR8) [11]. The activity of these enzymes results in the production of a hairpin structure precursor miRNA (pre-miRNA) in the nucleus. The pre-miRNAs are double-stranded, approximately 70 nucleotides in length and contain a terminal loop. The nuclear export factor exportin-5 then transfers pre-miRNA to the cytoplasm for additional processing by the RNase III enzyme (DICER) to create a mature miRNA:miRNA duplex without a hairpin structure (approximately 22 nucleotides long) [17]. In the last step of miRNA synthesis, miRNA duplexes are processed by a helicase into single-stranded miRNAs which are loaded onto the Argonaute (AGO) protein to form the multiprotein RNA-induced silencing complex (RISC) (Figure 1). In this complex, single-stranded miRNAs are able to affect gene expression. Generally, only one strand will be the single-stranded, mature miRNA, and the other strand will be degraded. Usually the miRNA strand with the thermodynamically less stable 5′ end is incorporated in the RISC complex [16,17,18]. In the RISC complex, miRNAs inhibit the translation of target mRNAs or promote their destabilization and degradation by imperfect sequence-specific binding to the 3′-UTR of target mRNAs [9,14,16,17].

To date, the most widely used approaches for investigating the impact of miRNAs in various biological conditions are systemic and organ-specific knockdowns/transgenic strategies, gain-of-function strategies, and loss-of-function strategies [19,20]. miRNA knockdowns are used for monitoring the specific function of selected miRNAs in the organism, where the deficiency of a miRNA is reflected in the changed expression of proteins and in the health of the organism. A good example of this approach was the first use of a miRNA to inhibit the expression of a specific membrane protein in the heart, called the sodium/calcium exchanger (NCX), in order to determine the functional significance of the sarcolemmal NCX in cardiac excitation-contraction coupling [21]. A specific miRNA, targeted to the NCX in isolated cardiomyocytes, knocked the expression of the NCX down by >90% and demonstrated that the NCX was important but not critical for cardiac contraction [21]. This miRNA approach can be much more effective than many other molecular strategies for the modification of gene and protein expression [22].

Gain-of-function strategies represent the injection or transfection of miRNA or synthetic miRNA mimics into tissue or cells, causing the overexpression of specific proteins by lentivirus or adeno-associated virus (AAV) during infection [20]. Alternatively, loss-of-function strategies include: (a) Application of anti-miRNAs (oligonucleotides capable of specifically binding and then inhibiting a target miRNA, which then leads to the downregulation of that miRNA) [5,23]; (b) application of miRNA sponges which contain a binding site for a miRNA family and which block miRNA activity [24,25]; and c) miRNA masking by oligonucleotides which hide the binding site of target mRNAs, leading to the prevention of degradation or inhibition of protein synthesis by miRNAs [26].

miRNAs can also be considered as new diagnostic markers. They have gained this attribute for three reasons: (a) Their high stability after isolation (at room temperature and also during multiple freeze–thaw cycles, probably due to their connection with AGO2 complexes, lipoproteins and their enrichment in circulating vesicles); (b) their presence in so many biological materials (plasma, serum, urine, saliva or seminal fluid); and (c) their significantly different expression between normal and pathological conditions. microRNA-based biomarkers have the ability to identify metabolic problems during disease latency (preclinical), assess the severity of a disease, identify the predisposition to a disease (assess risk), address disease etiology, confirm a diagnosis or reduce the incidence of a misdiagnosis on the basis of current clinical markers, and, finally, monitor the biological response to any experimental or clinical intervention. However, using miRNA as biomarkers of diseases requires a careful standardization of RNA manipulation methods like RNA isolation, detection and normalization, in order to obtain valuable, reliable information [6,27,28].

## 3. miRNAs and Cardiovascular Diseases

Many studies have detected a significant difference in the expression of miRNAs under different conditions, including various cardiovascular disorders [29,30,31,32]. This has led to the conclusion that miRNAs could be used as suitable biomarkers for CVD or as potential therapeutic targets. These changes in miRNAs were observed in both plasma and in cardiac tissue [6,33]. It is now clear that more than one miRNA can be involved in a single CVD or in different CVDs. The following summarizes the most recent data on the most important miRNAs identified to date which are involved in the most frequent CVDs. Changes of miRNAs were detected under disease conditions in patients or in animal models.

### 3.1. Cardiac Hypertrophy

Generally, cardiac hypertrophy develops as a compensatory mechanism in response to stressful stimuli. This may or may not ultimately lead to heart failure [6]. During hypertrophy, changes in miRNA expression were observed, mainly miRNA-1 and -133. These miRNAs are highly expressed in the heart and, according to Zhao and co-workers [34], their inhibition causes significant cardiac injury. MiRNA-1 affects cardiomyocyte growth and hypertrophy through inhibition of the calcineurin/NFAT (Nuclear factor of activated T cells) signaling pathway by regulating the expression of myocyte enhancer factor-2a (Mef2a) and GATA binding protein 4 (Gata4) [54]. Another possible target of miRNA-1 is twinfilin-1, an important cytoskeletal regulatory protein. Downregulation of miRNA-1 caused an upregulation of twinfilin-1 which led to a positive regulation of cardiac cytoskeletal cells [35]. Inhibition of miRNA-133 was observed in patients and animals with cardiac hypertrophy, probably by regulating anti-hypertrophic genes like guanosine triphosphate-guanosine diphosphate (GDP-GTP) exchange protein, or signal transduction kinase cell division control protein 42 (Cdc42) [36]. Other miRNAs that are associated with cardiac hypertrophy include miRNA-208, -21, -18b, -195, -199, -29, -22 and -23 [6,29,55].

### 3.2. Cardiac Arrhythmias

MicroRNA-1 and -133 are also involved in the pathology of arrhythmias. Increased expression of these miRNAs was found in arrhythmic hearts [38]. Both miRNA-1 and -133 modulated the expression of K^+^ channels (mostly K^+^/Na^+^ hyperpolarization-activated cyclic nucleotide-gated ion channel (HCN)-2 and HCN-4 located in the pacemaker [32,37,38]), but they also altered the expression of gap junction alpha-1 protein (GJA1) and potassium voltage-gated channel subfamily J member 2 protein (KCNJ2), affecting connexin43 and Kir2.1 expression [32,38,56]. Arrhythmogenic processes are also affected by miRNA-217-5p, -208, -499-5p, and -708-5p [39,57].

### 3.3. Cardiac Fibrosis

Cardiac fibrosis represents an important mechanism in the healing process and for adverse cardiac remodeling typical of many CVDs. Fibrosis is strictly regulated by many signaling pathways and factors but, under some conditions, excessive fibrosis can occur. The large accumulation of collagens (mostly collagen type I and type III) and other proteins of the extracellular matrix can lead to impaired cardiac contractility and the development of arrhythmias [6]. One of the most important miRNAs involved in the process of fibrosis is miRNA-21. This miRNA regulates survival of fibroblasts and secretion of growth factors by affecting the ERK-2 MAP (Mitogen activated protein) kinase pathway through inhibition of sprouty homologue 1 (Spry1) [40]. In the fibrotic mouse, upregulation of miRNA-21 was observed and its inhibition improved the level of fibrosis and heart function [40]. A significant change in the expression levels of miRNA-133, -15 family, -29 family, -26a, -24, and -590 have also been associated with cardiac fibrosis [6,29,41,42,43,44].

### 3.4. Coronary Artery Disease

Coronary artery disease (CAD) is one of the most common types of heart disease. The impaired blood flow in CAD leads to cardiac ischemia that, if severe enough, may cause an infarction. In CAD patients, an increase in the expression of miRNA-1, -21, or -208 has been detected. Decreases in the expression levels of miRNA-133, -126-3p, -195, -145, -17, and -155 have also been identified [31,33,45,46,58]. Interestingly, Dong and colleagues [59] have suggested that the highly expressed miRNA-126-3p levels observed in non-infarcted areas of rat hearts after an infarction may mean that this miRNA can play a significant role in the myocardial recovery after myocardial infarction [45,59]. Reddy et al. [49] and Schulte and Zeller [50] demonstrated significant association between increased levels of plasma miRNA-33 and coronary artery diseases. O´Sullivan et al. [47] reported that miRNA-93-5p is the most dysregulated miRNA in patients with CAD and may represent the strongest predictor of CAD in their study [47]. The downregulation of ATP-binding cassette A1 (ABCA1) by miRNA-93-5p has also been suggested to induce an increase in the circulating levels of cholesterol that may contribute to coronary atherosclerosis and CAD [60].

### 3.5. Heart Failure

Any of the pathologies discussed above could lead to the development of heart failure, a condition wherein the heart is unable to meet its circulatory demands. Many miRNAs are changed in models of heart failure, including miRNA-199b, -195, -100, -133, -24, and -208 [31,33,51]. MiRNA-199b (miR-199b) was increased during heart failure and appeared to target the calcineurin/NFAT pathway. MiRNA-199b targets the nuclear NFAT kinase dual-specificity tyrosine-(Y)-phosphorylation regulated kinase 1a (Dyrk1a) in a process that constitutes a pathogenic feed-forward mechanism affecting calcineurin-responsive gene expression. In vivo inhibition of miR-199b caused normalization of Dyrk1a expression, a reduction of nuclear NFAT activity and inhibition of hypertrophy and fibrosis in mouse models of heart failure [61]. Changed expressions of miRNAs miR-1, -214, -29b, -342, -7, -107, -126, -125, -122, -423-5p, -320a, -650, -1228, -662, -583, -3175, -21, -22 and miR-92b have been shown in other studies of heart failure [33,52,53].

## 4. Nutritional Aspects of Cardiovascular Diseases

The risk of CVD is substantially influenced by many factors, including diet. The Mediterranean diet is a good example of this as it has been associated with broad healthy benefits on human health. The Mediterranean diet represents a collection of eating habits traditionally followed by people in different countries bordering the Mediterranean Sea. It is characterized by a high intake of olive oil, fruit, nuts and seeds, vegetables, cereals, and a moderate intake of fish and red wine. Moderate intake of dairy products, as well as eggs, and chicken are allowed, whereas red meat is avoided [62].

**Figure 2:**
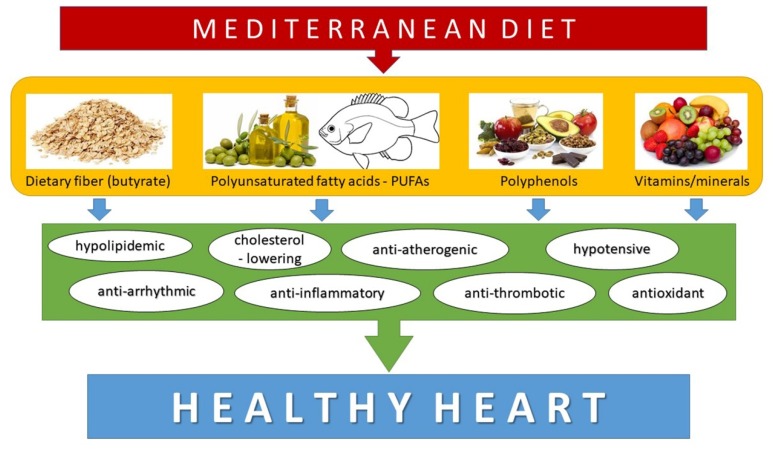
The main bioactive dietary components of the Mediterranean diet and their beneficial effects on the cardiovascular system.

The Mediterranean diet is particularly protective against CVD. Grosso et al. [63] reported a 25% lower risk of CVD mortality in people adhering to the Mediterranean diet. A meta-analysis of seven cohort studies showed that adherence to the Mediterranean diet was associated with a low risk of coronary heart disease [64]. Keys et al. [65] hypothesized that the Mediterranean diet exhibited protection against CVD and several other diseases principally because of its low saturated fat content. However, its protective effects can also be attributed to its rich content of the bioactive components olive oil, fruits, vegetables and legumes (Figure 2).

Olive oil is the main source of vegetable fat in the Mediterranean diet. It is mainly comprised of the mono-unsaturated fatty acid (MUFA) oleic acid. Olive oil also contains high amounts of bioactive compounds, including vitamin E, polyphenols (mainly flavonoids) and other minor phytochemicals [66]. Observational studies have suggested that olive oil intake is inversely associated with CVD, in both the Spanish general population [66] and in a cohort of Italian women [67]. Olive oil bioactive compounds exhibited a capability to attenuate oxidative stress and improved endothelial function through their anti-inflammatory, anti-oxidant and anti-thrombotic properties, thereby reducing the risk and progression of atherosclerosis [68]. The main phenolic compounds present in olive oil are hydroxytyrosol and oleuropein, which are both potent antioxidants and enzyme modulators [69]. A study in rats demonstrated that the hypotensive effect of olive oil is associated with its high oleic acid content [70].

The cardioprotective action of an increased intake of fruit and vegetables in the diet has been demonstrated in several studies. A meta-analysis of prospective studies revealed inverse associations between the intake of apples and pears, citrus fruits, green leafy vegetables, cruciferous vegetables and CVD and all-cause mortality [2]. A randomized controlled trial showed a statistically significant effect of fruit and vegetable consumption on both plasma antioxidant concentrations and blood pressure [71]. It is assumed that the healthy effect of vegetables and fruits can be attributed to dietary fibre, vitamins, phytochemicals and minerals in these food items [1]. The bioactive component of tomatoes, lycopene, exhibited significant antioxidant, hypolipidemic and anti-atherogenic effects [72]. The consumption of grapes may reduce the incidence of CVD due to several phytochemicals [73]. The reduced incidence of CVD after apple consumption is probably a result of the cholesterol-lowering effect of the fibre and polyphenols (catechin, epicatechin) contained in apples [74]. Citrus flavonoids like naringin and hesperidin exert antihypertensive, lipid-lowering, antioxidant and anti-inflammatory properties, which could explain their anti-atherogenic action [75].

Nuts and seeds are a good source of polyunsaturated fatty acids (PUFAs) (mostly linoleic and alpha-linolenic acid), rich in dietary fibre, minerals (potassium, calcium, magnesium, selenium), vitamins (folate, vitamin C and E) and other bioactive compounds (coenzyme Q10, phytosterols and polyphenols) [76]. There is substantial evidence showing that the intake of nuts and seeds provides protection against CVD. Consumption of peanuts and walnuts was associated with a 13% to 19% lower risk of total CVD, respectively, and a 15% to 23% lower risk of coronary heart disease, respectively [77]. The primary mechanism by which nuts protect against CVD is through the improvement of lipids and lipoprotein profile via a lowering of oxidative stress, inflammation and an improvement in endothelial function [78]. Flaxseed represents one of the richest plant sources of omega-3 fatty acids (alpha-linolenic acid). Several preclinical and clinical studies have shown beneficial cardioprotective effects of flaxseed supplementation. These are attributed to antihypertensive, antiatherogenic, cholesterol-lowering and anti-inflammatory action of flaxseed bioactive components [79].

Fish is recommended as a part of healthy diet because of its cardioprotective effects [80]. Panagiotakos et al. [81] demonstrated that long-term fish intake was associated with a better lipid profile, lower arterial blood pressure and improved blood glucose levels in elderly people from Mediterranean islands. Consumption of fatty fish has been suggested to reduce the risk of CVD, primarily due to their high levels of omega-3 fatty acids [82]. Omega-3 fatty acids (belonging to PUFAs) may improve cardiac function by their anti-inflammatory, antithrombotic, anti-triglyceridemic, anti-atherogenic, and anti-arrhythmic effects [83,84,85]. Vitamin D is highly abundant in different fish species. It has been found to regulate the expression of pro-inflammatory cytokines and adhesion molecules, thus represents a valuable component in the prevention of atherosclerosis [86]. Fatty fish represents a good dietary source of coenzyme Q10 which has been shown to be cardioprotective in atherosclerosis, hypertension and heart failure [87].

Cereals are widely used in Mediterranean countries as in other parts of the world [88] and whole grains consumption induce a beneficial effect on CVD morbidity and mortality. Aune et al. [2] demonstrated significant reductions in the risk for CVD, stroke and coronary heart disease in patients who consumed an increased amount of whole grains (90 g/day). A meta-analysis evaluating the value of whole grains showed a benefit in a series of prospective cohort studies, with a 21% reduction in CVD events and mortality. Eating whole grains decreased total cholesterol and LDL-cholesterol levels [89]. These effects are mainly attributable to the content of dietary fibre. Dietary fibre has a positive effect, probably due to lowering the amount of serum cholesterol in the blood by increasing the excretion of bile acids in feces [90]. It may also reduce body weight, which would decrease systolic and diastolic blood pressure [91].

One of the main characteristics of the traditional Mediterranean diet is the moderate intake of wine, particularly red wine. Red wine contains high amounts of polyphenolic compounds (quercetin and catechin) and other compounds which are thought to be beneficial for cardiovascular health. Due to its abundant content of polyphenols, wine intake is associated with a lowering of CVD risk [92].

## 5. The Modulation of miRNAs by Dietary Components in Cardiovascular Diseases

**Figure 3:**
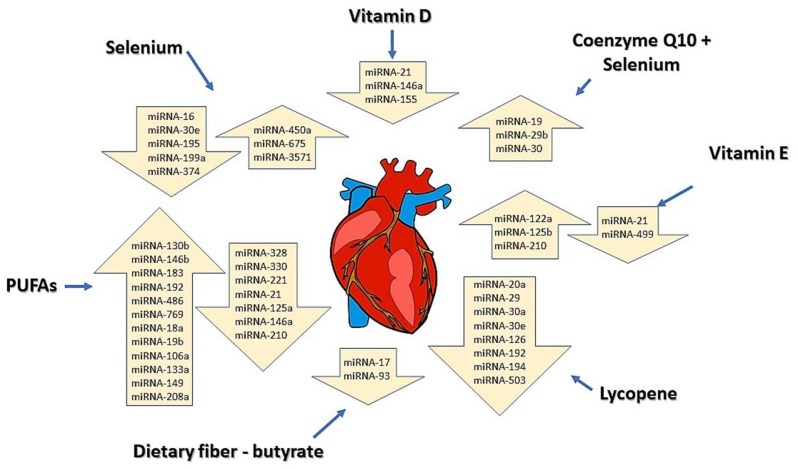
Selected bioactive dietary components and their modulation of miRNAs in the heart contribute to the prevention of CVD. In the arrows are placed miRNAs for which expression was changed in the diseased heart after individual nutrient administration. Upward arrows represent upregulation of miRNAs and downward arrows miRNAs which were downregulated. Changes in the expression of miRNAs shown in the figure were measured in CVD experimental models after administration of individual nutrients.

Many nutritional components modulate the expression of diverse miRNAs in different types of tissues and thereby influence whole body physiology [8,14,93]. These effects of diet on miRNAs may be very different than the changes in miRNAs shown in disease without dietary influence (Table 1). The summary of our current knowledge on the cardio-beneficial action of selected dietary compounds via miRNA modulation is presented in Figure 3. The effect of diet on selected miRNAs expression was examined under conditions of different CVDs.

Among cardioprotective nutritional components that can affect the expression of miRNAs are omega-3 and omega-6 PUFAs [94]. PUFAs were observed to downregulate miRNA-146a in endothelial cells with lipopolysaccharide-induced inflammation [95]. This miRNA can contribute to the induction of vascular inflammation [96]. Casas-Agustench et al. [97] observed that consumption of different kinds of fatty acids in pregnancy modulate the expression of miRNAs in both maternal and offspring tissues. Omega-3 PUFAs were able to reverse an angiotensin II-induced increase of miRNA-21 expression in mouse cardiac fibroblasts, therefore, they may exert potential beneficial effects in cardiac fibrosis [98]. miRNA-21 is also connected with CVD and inflammation [27]. Diets with a high content of PUFAs downregulate miRNA-21, which reduces pro-inflammatory signaling [99]. Downregulation of miRNA-21 after consumption of PUFAs was also observed in other studies of animal models and cell cultures [100,101]. Ma et al. [102] concluded that omega-3 PUFAs may have a protective effect on cardiomyocytes following myocardial infarction through their upregulation of anti-apoptotic miRNAs (miRNA-133a-5p, miRNA-149-5p, miRNA-208a-3p) and downregulation of pro-apoptotic miRNAs (miRNA-210-3p). Zheng et al. [94] reported that omega-3 PUFAs regulate miRNA-19b, -146b and -183 in Wistar rats. Administration of omega-3 PUFAs upregulated levels of these miRNAs and suppressed inflammatory markers compared with non-treated rats. In another study, Ortega et al. [103] observed an alteration in the levels of 11 miRNAs when 30 healthy people consumed 30 g of nuts/day (a food rich in PUFAs) for 8 weeks. The authors measured a downregulation of miRNA-328, -330, -221, and 125a and upregulation of miRNA-192, -486, -19b, -106a, -130b, -18a, and 769 after nut consumption.

Vitamins are essential micronutrients that have an important role in the prevention of CVD. Some recent studies suggest that vitamins may function through the regulation of miRNA expression [104,105,106]. Karkeni et al. [107] declared that vitamin D downregulates the expression of miRNA-146a and -155 in murine adipocytes through inhibiting NF-kB (Nuclear factor kappa-light chain-enhancer of activated B cells), ultimately leading to the suppression of inflammation. Deficiency of vitamin D is also connected with many pathological disorders such as hypertension [108], metabolic syndrome [109] and coronary artery disease [110]. Liu et al. [111] found that increased miRNA-21 affects vitamin D production through the inhibition of genes encoding enzyme 25(OH)D3-1α-hydrolase, which is important for the conversion of vitamin D from its inactive form to the active form. Sheane et al. [112] found a positive association between miRNA-21 expression and vitamin D deficiency in coronary artery disease. Studies with the effect of vitamin E (tocopherol) performed by Rimbach et al. [113] or by Gaedicke et al. [114] revealed upregulation of miRNA-122a and -125b in hepatic cells under vitamin E deficiency. Based on these results, the authors suggested that the consumption of vitamin E could be beneficial for human health because these miRNAs are mostly effective in lipid metabolism and inflammatory processes. Cohen et al. [115] demonstrated that vitamin E alleviates cardiac hypertrophy and fibrosis in mice via downregulation of miRNA-21 and -499 and upregulation of miRNA-210.

Lycopene is a carotenoid, found in red-colored fruits and vegetables. A beneficial effect against fibrosis was observed in the rat after administration of a tomato and its constituent lycopene after a myocardial infarction (MI). In this study, groups with tomato and lycopene supplementation experienced decreased interstitial fibrosis and improved diastolic dysfunction 3 months after an MI. They also observed a downregulated expression of 8 miRNAs after administration of lycopene – miRNA-29, -194, -503, -20a, -30a, -192, -30e, and 126. Based on these results, the authors suggested that the ingestion of lycopene could have a beneficial effect against MI through the modification of miRNA expression [116].

Alehagen et al. [117] reported that administration of selenium and coenzyme Q10 had an effect on many miRNAs. In this study, 443 healthy patients were administered selenium and coenzyme Q10 tablets for 4 years. The study found at least 70 miRNAs with significant differences in their expression compared to the placebo patients. Among them, the greatest difference in expression were miRNA-29b, miRNA-30, or miRNA-19, which have all been associated with CVD or cancer [117]. The effect of selenium on miRNA expression was also observed by Xing and colleagues [118] using a rat model of selenium deficiency. Selenium deficiency is a causative factor in heart failure. The authors identified five miRNAs which were extracted from the heart (miR-374, -16, -199a-5p, -195, and -30e*) that were upregulated >5-fold in the deficiency group, compared to the selenium-supplemented group. Other miRNAs (miR-3571, -675, and -450a*) were downregulated. The authors suggested that these miRNAs may regulate cardiac function.

Another nutrient with a beneficial effect on human health which may act through a modulation of miRNAs is dietary fiber. The study of Hu et al. [119] reported an association between butyrate, which is a metabolite of dietary fiber, and the expression of several miRNAs in human colon cancer cells (HCT-116). They observed decreasing expressions of 6 miRNAs in the presence of butyrate, including miRNA-17 and -93, miRNAs also widely expressed in the heart and changed in coronary artery disease [31,48,61].

Recent studies have suggested that gene expression could also be modulated by miRNAs present in the consumed food [8]. The most studied sources of miRNAs obtained from the diet are plant foods and cow milk. However, due to the existence of RNases and an unhospitable environment in the gastrointestinal tract, miRNAs which are received from dietary sources must be protected from degradation by internalization in exosomes or exosome-like structures [120]. Similar findings have been discussed by other authors [120,121] with the aim to use these formulations for pharmacological purposes against different diseases in humans.

## 6. Conclusions

Epidemiological studies indicate that nutrition influences the health status of humans. Prevention or even reversal of chronic diseases including CVD through diet is of high interest. Studies have revealed that individual bioactive nutrients are responsible for the cardioprotective effects of some dietary plans (e.g., the Mediterranean diet). However, the direct mechanisms of action are still not fully understood. Bioactive dietary components like PUFAs, vitamins, and minerals can be effective in CVD prevention and treatment due to their ability to change miRNAs expression, thereby modulating important pathways involved in lipid metabolism, endothelial function, hypertrophy and/or fibrosis. The capacity of food nutrients to modulate miRNAs involved in heart function and development (mainly miRNA-1, -21, -133 and -155) gives further rationale for the need for additional research to determine if these interactions between food and miRNAs can serve as viable targets for novel therapeutic approaches to CVD.

## Figures and Tables

**Table 1:** Summary of miRNAs dysregulated in different cardiovascular diseases (CVD).

CVD	Downregulated miRNAs	Upregulated miRNAs	References
Hypertrophy	miRNA-1, miRNA-133,	miRNA-208, miRNA-21, miRNA-29, miRNA-18b, miRNA-195, miRNA-199, miRNA-23, miRNA-22	[6,29,34,35,36]
Arrhythmias	miRNA-499-5p	miRNA-1, miRNA-133, miRNA-708-5p, miRNA-217-5p, miRNA-208	[32,37,38,39]
Fibrosis	miRNA-133, miRNA-29 family, miRNA-26a, miRNA-24, miRNA-590	miRNA-21, miRNA-15 family	[6,29,40,41,42,43,44]
Coronary artery diseases	miRNA-133, miRNA-126-3p, miRNA-195, miRNA-145, miRNA-17, miRNA-155, miRNA93-5p,	miRNA-1, miRNA-21, miRNA-208, miRNA-33	[31,33,45,46,47,48,49]
Heart failure	miRNA-126, miRNA-133, miRNA-1, miRNA-107, miRNA-3175, miRNA-583, miRNA-29b	miRNA-199b, miRNA-24, miRNA-208, miRNA-125, miRNA-195, miRNA-214, miRNA-423-5p, miRNA-320a, miRNA-22, miRNA92b, miRNA-122, miRNA-21, miRNA-650, miRNA-662, miRNA-1228, miRNA-100, miRNA-342	[33,50,51,52,53]
